# Tumor-promoting functions of transforming growth factor-β in progression of cancer

**DOI:** 10.3109/03009734.2011.638729

**Published:** 2012-04-19

**Authors:** Kohei Miyazono, Shogo Ehata, Daizo Koinuma

**Affiliations:** ^1^Department of Molecular Pathology, Graduate School of Medicine, University of Tokyo, Bunkyo-ku, Tokyo 113-0033, Japan; ^2^Ludwig Institute for Cancer Research, Box 595, SE 751-24 Uppsala, Sweden

**Keywords:** Angiogenesis, cancer-initiating cell, EMT, invasion, metastasis, TGF-β

## Abstract

Transforming growth factor-β (TGF-β) elicits both tumor-suppressive and tumor-promoting functions during cancer progression. Here, we describe the tumor-promoting functions of TGF-β and how these functions play a role in cancer progression. Normal epithelial cells undergo epithelial-mesenchymal transition (EMT) through the action of TGF-β, while treatment with TGF-β and fibroblast growth factor (FGF)-2 results in transdifferentiation into activated fibroblastic cells that are highly migratory, thereby facilitating cancer invasion and metastasis. TGF-β also induces EMT in tumor cells, which can be regulated by oncogenic and anti-oncogenic signals. In addition to EMT promotion, invasion and metastasis of cancer are facilitated by TGF-β through other mechanisms, such as regulation of cell survival, angiogenesis, and vascular integrity, and interaction with the tumor microenvironment. TGF-β also plays a critical role in regulating the cancer-initiating properties of certain types of cells, including glioma-initiating cells. These findings thus may be useful for establishing treatment strategies for advanced cancer by inhibiting TGF-β signaling.

## Introduction

Transforming growth factor-β (TGF-β) is a multifunctional regulator of cell growth, apoptosis, differentiation, and migration. TGF-β1 was originally discovered as a secreted protein that induces anchorage-independent growth in normal rat kidney NRK49F fibroblasts in the presence of epidermal growth factor (EGF) (1). TGF-β was shown to potently inhibit the proliferation of most cell types, including epithelial cells, endothelial cells, hematopoietic cells, and lymphocytes, and is widely known as a tumor suppressor. Studies investigating TGF-β signaling have revealed that perturbations of the TGF-β signaling pathway, such as mutations of TGF-β receptors or Smad proteins, lead to cancer progression and are related to poor prognosis of certain types of cancer. However, recent findings have shown that cancer cells become resistant to the growth inhibitory activity of TGF-β and that TGF-β facilitates invasion and metastasis of these cells both *in vitro* and *in vivo*.

Accumulating evidence has revealed that TGF-β plays a bidirectional role in cancer progression (2,3). TGF-β acts as a tumor suppressor by inhibiting cell growth through suppressing c-Myc expression and stimulating certain cyclin-dependent kinase inhibitors, including p21^WAF1^ and p15^Ink4b^, and by inducing cellular apoptosis through inducing DAP kinase, GADD45β, and Bim (4). Conversely, TGF-β functions as a tumor-promoting factor by stimulating extracellular matrix deposition and tissue fibrosis, perturbing immune and inflammatory function, stimulating angiogenesis, and promoting epithelial-mesenchymal transition (EMT).

In this review article, we discuss the tumor-promoting functions of TGF-β, particularly on EMT, on the basis of recent findings in our laboratory. We also describe the function of TGF-β in some cancer-initiating cells and discuss how inhibition of TGF-β signaling can be used for treating different types of cancer.

## TGF-β family signaling

**Figure 1.**
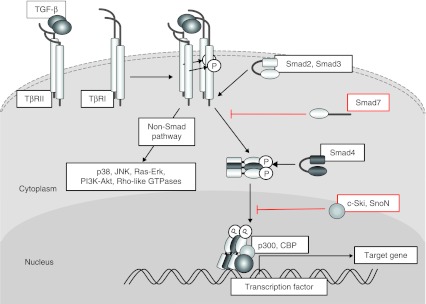
Schematic representation of TGF-β signal transduction pathways. TGF-β transduces signals through two different types of serine/threonine (and tyrosine) kinase receptors, termed TβRI and TβRII. Upon TGF-β binding, TβRI and TβRII form heterotetrameric complexes, and TβRII kinase transphosphorylates the juxtamembrane portion (GS domain) of the cytoplasmic region of TβRI. Phosphorylated TβRI transmits intracellular signaling through R-Smad phosphorylation. Smad2 and Smad3 are R-Smads phosphorylated by TβRI kinase and form heteromeric complexes with Smad4 (co-Smad). Smad complexes translocate into the nucleus and act as transcriptional regulators of target genes by interacting with other transcription factors and transcriptional regulators. Smad7 (I-Smad), which lacks the typical MH1 domain, interferes with the activation of R-Smads by interacting with TβRI and competitively prevents R-Smads from being phosphorylated by TβRI. TGF-β activates other intracellular signaling pathways in addition to Smads in order to regulate a wide array of cellular functions. These non-Smad pathways are activated by TGF-β receptors through phosphorylation or direct interaction.

TGF-β binds to two different serine/threonine kinase receptors, TβRII and TβRI (5). Betaglycan, also known as the TGF-β type III receptor, facilitates binding of TGF-β (particularly TGF-β2 among the three isoforms of TGF-β) to TβRII. TβRII activates TβRI through phosphorylation of the Gly-Ser-rich (GS) domain of TβRI, which in turn phosphorylates and activates Smad2 and Smad3, receptor-regulated Smads (R-Smads) specific for TGF-β and activin signaling (Figure 1). Bone morphogenetic proteins (BMPs) activate another set of R-Smads, including Smad1, Smad5, and Smad8 (6). Activated Smad2 and Smad3 form complexes with Smad4, common partner Smad (co-Smad), and translocate into the nucleus. R-Smad/co-Smad complexes associate with various transcription factors (AP-2, Ets, and HNF-4α (7–9)) and transcriptional co-activators (p300, CBP, and GCN5) or co-repressors (p107, Ski, and SnoN) in the nucleus and regulate transcription of a wide spectrum of TGF-β target genes. Smad7, an inhibitory Smad (I-Smad), represses TGF-β signaling through multiple mechanisms; among these mechanisms, binding to activated type I receptors and competition with R-Smads for receptor binding play a major role in regulation of TGF-β signaling (10). c-Ski (also known as SKI) and the related SnoN (also known as SKIL) bind directly to Smad2/3 and Smad4 and function as transcriptional co-repressors by recruiting histone deacetylases and competing for binding with p300/CBP. C-Ski also disrupts formation of the R-Smads and co-Smad complex to inhibit TGF-β signaling (11). In addition to its involvement in Smad signaling pathways, TGF-β activates various non-Smad signaling pathways, including ERK, JNK, and p38 MAP kinases, phosphatidylinositol-3 kinase (PI3K)-Akt, and small GTPase pathways (12). TβRI functions as a dual-specificity kinase (tyrosine and serine/threonine kinase) and phosphorylates ShcA on tyrosine and serine residues to activate the MAP kinase pathway (13).

## Induction of EMT

EMT is a differentiation switch through which epithelial cells differentiate into mesenchymal cells, and it occurs in the process of tissue morphogenesis during development, wound repair, and cancer progression in adult tissues (14,15). An early event of EMT includes disruption of tight junctions connecting epithelial cells and delocalization of tight junction proteins, such as ZO-1, claudin-1, and occludin. Early events of EMT also include disruption of adherence junctions, which contain E-cadherin and β-catenin, and reorganization of the actin cytoskeleton. Epithelial cells lose cell polarity and show spindle-like morphology with expression of various mesenchymal markers, including N-cadherin, fibronectin, and α-smooth muscle actin (α-SMA). Cell motility and invasive properties are enhanced in resulting mesenchymal cells.

EMT can be classified into three subtypes (16). Type 1 EMT occurs during development and includes the mesenchymal transition of primitive epithelial cells during gastrulation, generation of migrating neural crest cells from neuroepithelial cells, and formation of endocardial cushion tissue from cardiac endothelial cells. Type 2 EMT includes the transition of secondary epithelial (and endothelial) cells to tissue fibroblasts, which can be observed during the processes of wound healing, regeneration, and fibrosis in adult tissues. Type 3 EMT also occurs in adult tissues and involves the mesenchymal transition of epithelial carcinoma cells, leading to generation of metastatic tumor cells.

TGF-β is well known to induce EMT in various epithelial cells, including normal mouse epithelial NMuMG cells and A549 lung adenocarcinoma cells (17). Many transcription factors, including the two-handed zinc-finger factors δEF1 (also known as ZEB1) and SIP1 (ZEB2), the zinc-finger factors Snail (also known as SNAI1) and Slug (SNAI2), and the basic helix-loop-helix (bHLH) factors Twist and E12/E47, are induced by TGF-β signaling in a Smad-dependent fashion and play critical roles in EMT induction. Additionally, non-Smad signaling pathways activated by TGF-β and cross-talk with other signaling pathways, including fibroblast growth factor (FGF) and tumor necrosis factor-α (TNF-α) signaling, play important roles in EMT promotion.

## Induction of EMT in tumor stromal cells by TGF-β

**Figure 2.**
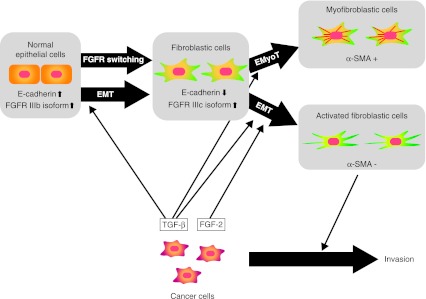
Schematic representation of EMT induction by TGF-β and FGF-2. ‘Epithelial cells’ differentiate into ‘fibroblastic cells’ through EMT induced by TGF-β and further differentiate into α-SMA-positive ‘myofibroblastic cells’ through epithelial-myofibroblastic transition (EMyoT). When FGF-2 is present in this process, FGF-2 induces differentiation of epithelial cells to ‘activated fibroblastic cells’.

Epithelial cells in the tumor stroma undergo EMT (type 2 EMT) and play a critical role in cancer progression. We cocultured NMuMG cells with mouse mammary tumor JygMC(A) cells and found that NMuMG cells that have undergone EMT express α-SMA (18). The effect of the JygMC(A) cells was abolished by treatment with the TβRI inhibitor SB431542. Interestingly, when NMuMG cells were cocultured with the mouse mammary tumor cell line 4T1, NMuMG cells underwent EMT and produced mesenchymal cells with an activated fibroblastic phenotype, which lacked α-SMA expression. 4T1 cells produced TGF-β1 at a level comparable to that produced by JygMC(A) cells. When 4T1 cells were treated with FGF receptor 1 (FGFR1) inhibitor SU5402, α-SMA-positive NMuMG cells were detected, indicating that the loss of α-SMA expression is due to FGF(s) secreted from 4T1 cells. We have shown that treatment of NMuMG cells with TGF-β and FGF-2 prevents the production of mesenchymal cells expressing α-SMA and calponin by activating the MEK-ERK pathway. Interestingly, NMuMG cells that have undergone EMT following treatment with TGF-β and FGF-2 exhibit drastic morphological changes with marked actin reorganization, enhanced cell migration, and increased production of matrix metalloproteinases (MMPs), including MMP-9. Moreover, NMuMG cells treated with TGF-β and FGF-2 enhanced the invasion of cocultured breast cancer cells into collagen gels *in vitro*. Thus, TGF-β and FGF-2 co-operate with each other to produce ‘activated’ fibroblasts in the tumor microenvironment, and activated fibroblasts may in turn secrete substances such as MMPs to induce invasion and metastasis of adjacent cancer cells (Figure 2).

During EMT progression, TGF-β induces isoform switching of FGFRs. Of the 22 FGFs (19), epithelial cells respond to specific FGFs, including FGF-7 (also known as keratinocyte growth factor (KGF)), but not to FGF-2 (basic FGF) or FGF-4. However, cells that have undergone EMT become responsive to FGF-2 and FGF-4, but not to FGF-7 (18). We have shown that TGF-β-mediated EMT induces isoform switching of FGFRs through alternative splicing, following which expression of the IIIb isoform of FGFR decreased and that of the IIIc isoform increased. Exon array analysis showed that TGF-β alters a broad spectrum of splicing patterns by reducing the expression of epithelial splicing regulatory proteins (ESRPs) 1 and 2 (20). Warzecha et al. (21) recently reported that the ESRP-regulated splicing pathway is abrogated during EMT. We found that repression of the expression of ESRPs by TGF-β is mediated by up-regulation of the δEF1 family proteins δEF1 and SIP1, which suppress the transcription of ESRP(s) by binding to the ESRP promoter(s). Interestingly, the expression profiles of ESRPs were reciprocally correlated with those of δEF1 and SIP1 in human breast cancer cell lines as well as in tumor specimens. In addition to FGFRs, TGF-β induces alternative splicing of CD44, Mena, and CTNND1 (also known as δ-catenin or p120 catenin), which are reportedly involved in cancer progression. We have also shown that over-expression of ESRPs attenuates TGF-β-induced EMT and restores the expression of E-cadherin and some other epithelial phenotypes. Thus, ESRPs are downstream targets of TGF-β and serve as antagonists to EMT by regulating alternative splicing of specific genes involved in TGF-β-induced EMT.

## Induction of EMT in cancer cells

EMT is observed in some transformed epithelial cells (type 3 EMT) to facilitate their invasive and metastatic properties. Type 3 EMT can be regulated by specific oncogenic and anti-oncogenic signals. We have shown that a zinc-finger transcription factor Snail is induced by TGF-β in pancreatic cancer Panc1 cells and plays a key role in EMT progression (22). Panc1 cells express active *K-ras*, and we found that induction of Snail by TGF-β is dependent on oncogenic Ras signals. Snail was strongly induced by TGF-β in Panc1 cells, but knock-down of Ras in Panc1 cells abolished Snail induction by TGF-β. Consequently, TGF-β failed to efficiently induce EMT in Panc1 cells in the absence of active Ras signaling. Exogenous expression of constitutively active Ras into HeLa cells resulted in marked induction of Snail by TGF-β, while induction of other direct targets of TGF-β, including Smad7 and PAI-1, was not enhanced by Ras signaling. MAP kinases have been reported to phosphorylate the linker region of Smad2 and Smad3, which both positively and negatively regulates TGF-β signaling (23). However, MAP kinase signaling was not required for induction of Snail by TGF-β, and it is currently unknown which downstream signals of Ras co-operate with TGF-β signaling (22).

Thyroid transcription factor-1 (TTF-1, the protein product of the *NKX2.1* gene) is expressed in normal lung tissues and acts as a master regulator of lung morphogenesis (24). TTF-1 is primarily expressed in type II pneumocytes and Clara cells and frequently expressed in lung cancer cells, including lung adenocarcinoma cells. Although the *TTF1* gene is amplified in some lung adenocarcinoma cells and may function as an oncogene (25), loss of TTF-1 expression is reportedly associated with poor prognosis of lung carcinoma. Recently, Winslow et al. (26) reported that TTF-1 controls differentiation of lung carcinoma cells and limits their metastatic potential in mice with active K-Ras and inactive p53. Interestingly, we found that TTF-1 functions as a tumor-suppressor during EMT induction. TTF-1 is highly expressed in certain types of lung adenocarcinoma cell lines, including H441 cells and LC-2/ad cells, but not in A549 cells (27). A549 cells show a spindle-like phenotype and grow rapidly, while H441 cells show tight cell–cell junctions with cobblestone-like morphology and grow much more slowly than A549 cells. A549 cells express low levels of TTF-1 and E-cadherin, while H441 cells express high levels of TTF-1 and E-cadherin. We have further shown that exogenous expression of TTF-1 in A549 cells inhibits TGF-β-induced EMT, decreases MMP-2 activity, cell migration, and cellular invasive capacity, and restores the epithelial phenotype through high E-cadherin expression. Conversely, TGF-β induces the expression of Snail and Slug in A549 cells, and silencing of TTF-1 in H441 cells enhances TGF-β-mediated EMT. TTF-1 has been reported to interact physically with Smad3 (28) and may inhibit Smad3 function. We have also shown that TGF-β down-regulates TTF-1 expression in A549 cells and that TTF-1 inhibits the expression of TGF-β2, which is expressed in epithelial cells at the tip of the distal airway during lung morphogenesis. Thus, TTF-1 may exert a tumor-suppressive effect through antagonizing the effect of TGF-β. These findings indicate a functionally inverse relationship between TTF-1 and TGF-β signaling in the progression of lung adenocarcinoma through regulation of EMT.

## TGF-β signaling in vascular tissues and angiogenesis

New blood vessel formation in tumor tissues (tumor angiogenesis) is essential for the growth and metastasis of tumor cells. Although TGF-β potently inhibits the growth of endothelial cells *in vitro*, it functions as a pro-angiogenic factor and stimulates angiogenesis *in vivo*. Increased expression of TGF-β is correlated to increased vascular density in some types of tumors.

For induction of tumor angiogenesis, TGF-β induces the expression of angiogenic factors, including connective tissue growth factor (CTGF) and vascular endothelial growth factor (VEGF) (29). Additionally, TGF-β stimulates the synthesis of MMP-2 and MMP-9 and down-regulates the expression of tissue inhibitors of metalloproteinase (TIMPs) in tumor tissues. Increased MMP activity leads to stimulation of migration and invasion of vascular endothelial cells, resulting in accelerated tumor angiogenesis.

However, TGF-β suppresses angiogenesis in certain types of tumors through reduced expression of some angiogenic factors or increased expression of angiogenic inhibitors. In diffuse-type gastric carcinoma, TGF-β induces the production of some angiogenic inhibitors, including thrombospondin-1 and TIMP-2, and perturbations of TGF-β signaling may thus lead to induction of angiogenesis and tumor growth *in vivo* (30,31).

In addition to induction of tumor angiogenesis, TGF-β acts on vascular endothelial cells and may disrupt cell–cell junctions and support the colonization of tumor cells to establish metastasis. Using endothelial cells derived from mouse embryonic stem (ES) cells, we showed that TGF-β suppresses the expression of claudin-5 and disrupts sheet formation *in vitro* (32). We also showed that TGF-β induces differentiation of certain endothelial cells into mesenchymal cells, resulting in the loss of tight cell–cell contacts *in vitro* (33). Moreover, through disruption of endothelial cell–cell junctions by inducing angiopoietin-like 4 (Angptl4) expression, TGF-β has been shown to increase the permeability of blood vessels and stimulate the trans-endothelial movement of cancer cells (34).

## Acceleration of cancer metastasis by TGF-β signaling

TGF-β facilitates metastasis of certain types of cancer in advanced stages, including breast cancer (35). Inhibition of TGF-β signaling may thus be a potential strategy for preventing metastasis of advanced cancers. Though not discussed in detail in this review, TGF-β regulates tumor development by regulating immune functions (36,37). Wakefield and colleagues reported that inhibition of TGF-β function prevents the progression of breast cancer by enhancing various immune functions (38).

**Figure 3.**
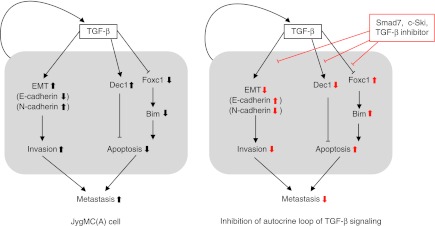
Mechanisms of TGF-β action on prevention of breast cancer metastasis using JygMC(A) cells. Endogenously activated autocrine loop of TGF-β regulates the expression of E-cadherin and N-cadherin by inducing EMT in JygMC(A) cells. Autocrine TGF-β also regulates the expression of various transcription factors, including Dec1 and Foxc1, and promotes the survival. Negative regulators of TGF-β signaling (Smad7, c-Ski, or TGF-β inhibitors) block these pathways and inhibit metastasis of JygMC(A) cells.

We have shown that Smad7, an I-Smad that inhibits TGF-β and BMP signaling, efficiently inhibits lung and liver metastasis of mouse breast cancer JygMC(A) cells (39). We subcutaneously inoculated JygMC(A) cells, which spontaneously metastasize to the lung, liver, and other organs in 3 to 4 weeks, in nude mice. Ten days after subcutaneous inoculation, adenoviruses containing Smad7 or LacZ were intravenously administered to the mice once weekly. Mice bearing JygMC(A) tumors and treated with LacZ adenovirus developed numerous metastases to the lung and liver, and all mice died by 50 days (median survival time, 41 days) after inoculation of JygMC(A) cells. In contrast, mice treated with Smad7 adenovirus showed a significant decrease in metastases of tumors in both the lung and liver, and the median survival time of Smad7-treated mice was 55 days. JygMC(A) cells treated with Smad7 showed increased expression of components involved in adherence and tight junctions, including E-cadherin, and decreased expression of mesenchymal markers, including N-cadherin. Smad7 also inhibited the migration and invasion of cells, indicating that Smad7 leads to prevention of the EMT process. Interestingly, Smad6, which preferentially inhibits BMP signaling, failed to show significant effects on the metastasis of JygMC(A) cells in nude mice, whereas c-Ski adenovirus showed effects similar to Smad7. Thus, inhibiting TGF-β signaling using Smad7 or c-Ski prevents the EMT process and eventually inhibits lung and liver metastasis of JygMC(A) cells (Figure 3).

In addition to preventing EMT, TGF-β appears to inhibit metastasis of JygMC(A) cells by some other mechanisms. Although TGF-β induces apoptosis of many different types of cells by inducing specific genes, it stimulates survival of certain types of cells in a context-dependent manner through activation of the PI3K-Akt signaling pathway. We have identified Dec1 (differentially expressed in chondrocytes 1, also known as SHARP2 and Stra13) as a downstream target of TGF-β–Smad signaling by DNA microarray analysis (40). Dec1 is a bHLH transcription factor, which is widely expressed in many tissues and over-expressed in certain types of cancer cells. Dec1 prevented the apoptosis of JygMC(A) as well as 4T1 cells, and a dominant-negative mutant of Dec1 suppressed lung and liver metastases of JygMC(A) cells in nude mice (Figure 3). Dec1 has been reported to induce the expression of an anti-apoptotic protein, survivin, in certain types of cells (41); however, we failed to show induction of survivin by TGF-β in JygMC(A) cells. Mechanisms of Dec1 induction of cell survival in JygMC(A) cells should be examined in the future.

We also found that inhibiting endogenous TGF-β signaling by a TβRI inhibitor, SB431542, induces the expression of the BH3-only protein, Bim (also known as Bcl2-like 11), in JygMC(A) and stimulates apoptosis in these cells (42). We showed that suppression of Bim expression by TGF-β is mediated by repression of a FOX family transcription factor, Foxc1, in JygMC(A) cells, thus suggesting an important role of the TGF-β–Foxc1–Bim axis in the survival of certain types of cells. Further studies are needed to determine whether the TGF-β–Foxc1–Bim axis is involved in lung and liver metastases of this type of cancer (Figure 3).

TGF-β also plays critical roles in bone metastasis, during which functional interaction between cancer cells and the bone microenvironment is important. *In-vivo* experimental models using intracardiac injection of cancer cells have been widely used to study the mechanisms of bone metastasis. Several studies revealed that TGF-β and its target molecules, such as parathyroid hormone-related protein (PTHrP) and interleukin-11 (IL-11), play critical roles in the development of bone metastasis of breast cancers (29,43), which occurs in a Smad-dependent fashion (44). PTHrP stimulates the expression of the RANK ligand (RANKL) in osteoblasts and induces differentiation of osteoclast precursors and resorption of bone. We studied the effects of a TβRI inhibitor, Ki26894, on bone metastasis in the human breast cancer cell line MDA-MB-231–5a-D (MDA-231-D), which is a highly metastatic variant of MDA-MB-231 cells. Ki26894 suppressed induction of PTHrP and IL-11 mRNA in MDA-231-D cells stimulated by TGF-β (45). When MDA-231-D cells were injected into the left ventricle of nude mice and treated with systemic administration of Ki26894 (treatment with Ki26894 was started 1 day before tumor cell inoculation), X-ray radiography showed that treatment with Ki26894 decreased bone metastasis of breast cancer cells and prolonged the survival of MDA-231-D-bearing mice compared to vehicle treatment. These findings suggest that inhibition of TGF-β signaling may be useful for preventing bone metastasis of advanced breast cancers.

## TGF-β maintains stemness of certain cancer-initiating cells

Cancer-initiating cells show increased tumor-initiating ability and often exhibit stem cell-like properties such as self-renewal, multipotency, and expression of specific stem cell markers. The concept of cancer-initiating cells reveals a new strategy of therapy against intractable cancers, though it remains unclear how cancer-initiating cells can be specifically eradicated. It is important to investigate the differences between cancer-initiating cells and normal stem cells and to identify specific molecules to target cancer-initiating cells without affecting the function of normal stem cells. Recent studies have also revealed critical roles of TGF-β signaling in the maintenance of stem cell-like properties of certain cancer-initiating cells, including glioma-initiating cells (GICs) (46,47), breast cancer-initiating cells (48), and leukemia-initiating cells in chronic myeloid leukemia (CML) (49).

**Figure 4.**
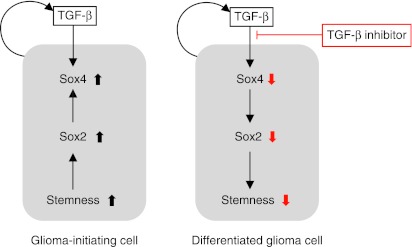
Effects of the TGF-β–Smad–Sox4–Sox2 axis on the maintenance of GIC stemness. TGF-β directly induces Sox4 expression. Subsequently, Sox4 promotes Sox2 expression, which plays significant roles in sustaining GIC stemness. TGF-β inhibitor blocks this TGF-β–Sox4–Sox2 axis, promotes GIC differentiation, and deprives these cells of their aggressiveness. Differentiated glioma cells (right panel) may be more sensitive to conventional chemotherapy and radiotherapy than undifferentiated GICs (left panel).

Glioma cells produce TGF-β1 and TGF-β2, and autocrine TGF-β signaling plays a pivotal role in maintaining the stem cell-like properties and tumorigenic activity of GICs (46,47). GICs obtained from patients with glioblastoma multiforme exhibit sphere-forming ability in a self-renewal medium containing EGF and FGF-2. Although TGF-β did not significantly affect the sphere-forming ability of GICs, a TβRI inhibitor, SB431542, efficiently reduced this ability in GICs. Moreover, SB431542 dramatically reduced the number of CD133-expressing cells and induced differentiation of GICs, leading to the appearance of cells expressing neural or glial cell markers. Analyses of TGF-β target genes using quantitative RT-PCR and by searching public datasets showed that TGF-β induces expression of the Sry-related HMG box (Sox) transcription factors Sox2 and Sox4. We showed that Sox4 is a direct target of Smad proteins activated by TGF-β and that it induces the expression of Sox2, which plays a critical role in the maintenance of GIC stemness. We also confirmed that in intracranial transplantation assays using immunocompromised mice, GICs pretreated with SB431542 showed decreased lethal potency. These results indicate that the TGF-β–Sox4–Sox2 pathway is essential for retaining the stemness of GICs, and inhibition of TGF-β signaling may be a potential method for treating glioma through targeting GICs (Figure 4).

Westermark and his colleagues (50) reported that another Sox family protein, Sox21, is expressed in glioma cells. Sox21 is an antagonizing partner of Sox2 and negatively regulates the expression of Sox2 in glioma cells. They showed that reduction in Sox2 expression using Sox2 siRNA or Sox21 over-expression reduced the cell number by inducing apoptosis.

In addition to the Sox4-Sox2 pathway, TGF-β also induces the expression of leukemia inhibitory factor (LIF) in a Smad-dependent fashion. LIF activates the downstream JAK-STAT pathway, leading to increased tumorigenesis of GICs (47). Anido et al. have shown that TGF-β inhibitors target GICs with high levels of CD44 and Id1 and that CD44^high^/Id1^high^ GICs are generally localized in the perivascular niche (51).

## Conclusion and perspectives

As proposed by Roberts and Wakefield (2), it is now well known that TGF-β exhibits both positive and negative effects on cancer progression. The bidirectional roles of TGF-β can be observed at the molecular, cellular, and tissue levels. Although we described the positive effects of TGF-β on maintaining the stemness of cancer-initiating cells, TGF-β has also been shown to decrease the number of specific types of cancer-initiating cells, including diffuse-type gastric carcinoma cells (52). Moreover, TGF-β induces maintenance of stem cell-like properties of certain breast cancer-initiating cells (48), while suppression of the TGF-β pathway leads to an increase in breast cancer-initiating cells in other types of breast cancer cells (53), thereby suggesting that the response to TGF-β varies depending on the type of cancer-initiating cells.

Mani et al. (48) reported that TGF-β maintains stem cell-like properties of certain cancer-initiating cells through induction of EMT. They showed that normal and transformed mammary epithelial cells acquired stem cell-like properties with high tumorigenic activity when EMT was induced in these cells by TGF-β. Although we have not determined whether the sizes of cancer-initiating cell compartments are affected by EMT in the pancreatic carcinoma Panc1 cells and lung adenocarcinoma A549 cells described above, these findings suggest a functional connection between EMT and cancer-initiating properties of certain epithelial cells.

Recent findings based on genome-wide analyses of Smad-binding sites in some types of cells, which were performed using ChIP-sequencing analyses, revealed that the binding profiles of Smads differ remarkably depending on the cell types and are affected by interaction with transcription factors expressed in each cell type and by cell-specific differences in baseline chromatin accessibility patterns (7,9,54). It is thus possible that the response of cells to TGF-β may be differentially affected by coexisting transcription factors and chromatin assembly patterns. Further studies examining global gene expression profiles and genome-wide maps of protein binding sites or epigenetic marks using high-throughput sequencing may be valuable for elucidating the mechanisms of differential cellular responsiveness to TGF-β.

## References

[1] Moses HL, Roberts AB (2009). The discovery of TGF-β: a historical perspective. The TGF-β family.

[2] Roberts AB, Wakefield LM (2003). The two faces of transforming growth factor-β in carcinogenesis. Proc Natl Acad Sci USA.

[3] Ikushima H, Miyazono K (2010). TGF-β signalling: a complex web in cancer progression. Nat Rev Cancer.

[4] Sanchez-Capelo A (2005). Dual role for TGF-β1 in apoptosis. Cytokine Growth Factor Rev.

[5] Heldin CH, Miyazono K, ten Dijke P (1997). TGF-β signalling from cell membrane to nucleus through SMAD proteins. Nature.

[6] Miyazono K, Kamiya Y, Morikawa M (2010). Bone morphogenetic protein receptors and signal transduction. J Biochem.

[7] Koinuma D, Tsutsumi S, Kamimura N, Taniguchi H, Miyazawa K, Sunamura M (2009). Chromatin immunoprecipitation on microarray analysis of Smad2/3 binding sites reveals roles of ETS1 and TFAP2A in transforming growth factor β signaling. Mol Cell Biol.

[8] Koinuma D, Tsutsumi S, Kamimura N, Imamura T, Aburatani H, Miyazono K (2009). Promoter-wide analysis of Smad4 binding sites in human epithelial cells. Cancer Sci.

[9] Mizutani A, Koinuma D, Tsutsumi S, Kamimura N, Morikawa M, Suzuki HI (2011). Cell type-specific target selection by combinatorial binding of Smad2/3 proteins and hepatocyte nuclear factor 4α in HepG2 cells. J Biol Chem.

[10] Kamiya Y, Miyazono K, Miyazawa K (2010). Smad7 inhibits transforming growth factor-β family type I receptors through two distinct modes of interaction. J Biol Chem.

[11] Deheuninck J, Luo K (2009). Ski and SnoN, potent negative regulators of TGF-β signaling. Cell Res.

[12] Moustakas A, Heldin CH (2005). Non-Smad TGF-β signals. J Cell Sci.

[13] Lee MK, Pardoux C, Hall MC, Lee PS, Warburton D, Qing J (2007). TGF-β activates Erk MAP kinase signalling through direct phosphorylation of ShcA. EMBO J.

[14] Thiery JP, Acloque H, Huang RY, Nieto MA (2009). Epithelial-mesenchymal transitions in development and disease. Cell.

[15] Sabe H (2011). Cancer early dissemination: cancerous epithelial-mesenchymal transdifferentiation and transforming growth factor-β signalling. J Biochem.

[16] Zeisberg M, Neilson EG (2009). Biomarkers for epithelial-mesenchymal transitions. J Clin Invest.

[17] Miyazono K (2009). Transforming growth factor-β signaling in epithelial-mesenchymal transition and progression of cancer. Proc Jpn Acad Ser B Phys Biol Sci.

[18] Shirakihara T, Horiguchi K, Miyazawa K, Ehata S, Shibata T, Morita I (2011). TGF-β regulates isoform switching of FGF receptors and epithelial-mesenchymal transition. EMBO J.

[19] Itoh N, Ornitz DM (2011). Fibroblast growth factors: from molecular evolution to roles in development, metabolism and disease. J Biochem.

[20] Horiguchi K, Sakamoto K, Koinuma D, Semba K, Inoue A, Inoue S TGF-β drives epithelial-mesenchymal transition through δEF1-mediated downregulation of ESRP. Oncogene.

[21] Warzecha CC, Jiang P, Amirikian K, Dittmar KA, Lu H, Shen S (2010). An ESRP-regulated splicing programme is abrogated during the epithelial-mesenchymal transition. EMBO J.

[22] Horiguchi K, Shirakihara T, Nakano A, Imamura T, Miyazono K, Saitoh M (2009). Role of Ras signaling in the induction of snail by transforming growth factor-β. J Biol Chem.

[23] Matsuzaki K Smad phosphoisoform signals in acute and chronic liver injury: similarities and differences between epithelial and mesenchymal cells. Cell Tissue Res.

[24] Minoo P, Su G, Drum H, Bringas P, Kimura S (1999). Defects in tracheoesophageal and lung morphogenesis in Nkx2.1-/- mouse embryos. Dev Biol.

[25] Weir BA, Woo MS, Getz G, Perner S, Ding L, Beroukhim R (2007). Characterizing the cancer genome in lung adenocarcinoma. Nature.

[26] Winslow MM, Dayton TL, Verhaak RG, Kim-Kiselak C, Snyder EL, Feldser DM (2011). Suppression of lung adenocarcinoma progression by Nkx2-1. Nature.

[27] Saito RA, Watabe T, Horiguchi K, Kohyama T, Saitoh M, Nagase T (2009). Thyroid transcription factor-1 inhibits transforming growth factor-β-mediated epithelial-to-mesenchymal transition in lung adenocarcinoma cells. Cancer Res.

[28] Minoo P, Hu L, Zhu N, Borok Z, Bellusci S, Groffen J (2008). SMAD3 prevents binding of NKX2.1 and FOXA1 to the SpB promoter through its MH1 and MH2 domains. Nucleic Acids Res.

[29] Kang Y, Siegel PM, Shu W, Drobnjak M, Kakonen SM, Cordon-Cardo C (2003). A multigenic program mediating breast cancer metastasis to bone. Cancer Cell.

[30] Komuro A, Yashiro M, Iwata C, Morishita Y, Johansson E, Matsumoto Y (2009). Diffuse-type gastric carcinoma: progression, angiogenesis, and transforming growth factor β signaling. J Natl Cancer Inst.

[31] Johansson E, Komuro A, Iwata C, Hagiwara A, Fuse Y, Watanabe A (2010). Exogenous introduction of tissue inhibitor of metalloproteinase 2 reduces accelerated growth of TGF-β-disrupted diffuse-type gastric carcinoma. Cancer Sci.

[32] Watabe T, Nishihara A, Mishima K, Yamashita J, Shimizu K, Miyazawa K (2003). TGF-β receptor kinase inhibitor enhances growth and integrity of embryonic stem cell-derived endothelial cells. J Cell Biol.

[33] Kokudo T, Suzuki Y, Yoshimatsu Y, Yamazaki T, Watabe T, Miyazono K (2008). Snail is required for TGF-β-induced endothelial-mesenchymal transition of embryonic stem cell-derived endothelial cells. J Cell Sci.

[34] Padua D, Zhang XH, Wang Q, Nadal C, Gerald WL, Gomis RR (2008). TGFβ primes breast tumors for lung metastasis seeding through angiopoietin-like 4. Cell.

[35] Yang YA, Dukhanina O, Tang B, Mamura M, Letterio JJ, MacGregor J (2002). Lifetime exposure to a soluble TGF-β antagonist protects mice against metastasis without adverse side effects. J Clin Invest.

[36] Yoshimura A, Wakabayashi Y, Mori T (2010). Cellular and molecular basis for the regulation of inflammation by TGF-β. J Biochem.

[37] Flavell RA, Sanjabi S, Wrzesinski SH, Licona-Limon P (2010). The polarization of immune cells in the tumour environment by TGF-β. Nat Rev Immunol.

[38] Nam JS, Terabe M, Mamura M, Kang MJ, Chae H, Stuelten C (2008). An anti-transforming growth factor-β antibody suppresses metastasis via cooperative effects on multiple cell compartments. Cancer Res.

[39] Azuma H, Ehata S, Miyazaki H, Watabe T, Maruyama O, Imamura T (2005). Effect of Smad7 expression on metastasis of mouse mammary carcinoma JygMC(A) cells. J Natl Cancer Inst.

[40] Ehata S, Hanyu A, Hayashi M, Aburatani H, Kato Y, Fujime M (2007). Transforming growth factor-β promotes survival of mammary carcinoma cells through induction of antiapoptotic transcription factor DEC1. Cancer Res.

[41] Li Y, Xie M, Yang J, Yang D, Deng R, Wan Y (2006). The expression of antiapoptotic protein survivin is transcriptionally upregulated by DEC1 primarily through multiple Sp1 binding sites in the proximal promoter. Oncogene.

[42] Hoshino Y, Katsuno Y, Ehata S, Miyazono K (2011). Autocrine TGF-β protects breast cancer cells from apoptosis through reduction of BH3-only protein, Bim. J Biochem.

[43] Yin JJ, Selander K, Chirgwin JM, Dallas M, Grubbs BG, Wieser R (1999). TGF-β signaling blockade inhibits PTHrP secretion by breast cancer cells and bone metastases development. J Clin Invest.

[44] Deckers M, van Dinther M, Buijs J, Que I, Lowik C, van der Pluijm G (2006). The tumor suppressor Smad4 is required for transforming growth factor β-induced epithelial to mesenchymal transition and bone metastasis of breast cancer cells. Cancer Res.

[45] Ehata S, Hanyu A, Fujime M, Katsuno Y, Fukunaga E, Goto K (2007). Ki26894, a novel transforming growth factor-β type I receptor kinase inhibitor, inhibits in vitro invasion and in vivo bone metastasis of a human breast cancer cell line. Cancer Sci.

[46] Ikushima H, Todo T, Ino Y, Takahashi M, Miyazawa K, Miyazono K (2009). Autocrine TGF-β signaling maintains tumorigenicity of glioma-initiating cells through Sry-related HMG-box factors. Cell Stem Cell.

[47] Penuelas S, Anido J, Prieto-Sanchez RM, Folch G, Barba I, Cuartas I (2009). TGF-β increases glioma-initiating cell self-renewal through the induction of LIF in human glioblastoma. Cancer Cell.

[48] Mani SA, Guo W, Liao MJ, Eaton EN, Ayyanan A, Zhou AY (2008). The epithelial-mesenchymal transition generates cells with properties of stem cells. Cell.

[49] Naka K, Hoshii T, Muraguchi T, Tadokoro Y, Ooshio T, Kondo Y (2010). TGF-β-FOXO signalling maintains leukaemia-initiating cells in chronic myeloid leukaemia. Nature.

[50] Ferletta M, Caglayan D, Mokvist L, Jiang Y, Kastemar M, Uhrbom L (2011). Forced expression of Sox21 inhibits Sox2 and induces apoptosis in human glioma cells. Int J Cancer.

[51] Anido J, Saez-Borderias A, Gonzalez-Junca A, Rodon L, Folch G, Carmona MA (2010). TGF-β receptor inhibitors target the CD44(high)/Id1(high) glioma-initiating cell population in human glioblastoma. Cancer Cell.

[52] Ehata S, Johansson E, Katayama R, Koike S, Watanabe A, Hoshino Y (2011). Transforming growth factor-β decreases the cancer-initiating cell population within diffuse-type gastric carcinoma cells. Oncogene.

[53] Tang B, Yoo N, Vu M, Mamura M, Nam JS, Ooshima A (2007). Transforming growth factor-β can suppress tumorigenesis through effects on the putative cancer stem or early progenitor cell and committed progeny in a breast cancer xenograft model. Cancer Res.

[54] Morikawa M, Koinuma D, Tsutsumi S, Vasilaki E, Kanki Y, Heldin CH (2011). ChIP-seq reveals cell type-specific binding patterns of BMP-specific Smads and a novel binding motif. Nucleic Acids Res.

